# Cell Atavistic Transition: Paired Box 2 Re-Expression Occurs in Mature Tubular Epithelial Cells during Acute Kidney Injury and Is Regulated by Angiotensin II

**DOI:** 10.1371/journal.pone.0093563

**Published:** 2014-04-07

**Authors:** Yushen Jiang, Tang Jiang, Juan Ouyang, Qingsong Zhou, Yanlan Liang, Yingpeng Cui, Peisong Chen, Bin Huang

**Affiliations:** 1 Department of Laboratory Medicine, The First Affiliated Hospital of Sun Yat-sen University, Guangzhou, Guangdong, China; 2 Department of Laboratory Medicine, The Third Affiliated Hospital of Sun Yat-sen University, Guangzhou, Guangdong, China; Queen Mary University of London, United Kingdom

## Abstract

The regeneration of tubular epithelial cells (TECs) after acute kidney injury (AKI) is crucial for the recovery of renal structure and function. The mechanism by which quiescent TECs re-obtain a potential to regenerate remains unknown. In this study, we observed a transient re-expression of embryonic gene Paired box 2 (Pax2) in adult rat TECs in vivo during ischemia-reperfusion induced AKI and most Pax2 positive TECs co-expressed kidney injury molecule-1 (KIM-1), a tubular injury marker. The re-expression of Pax2 was accompanied by increased levels of intrarenal Angiotensin II, which is a crucial injury factor of AKI. Furthermore, we also found a temporary re-expression of Pax2 in NRK-52E cells under the stimulation of Angiotensin II. This stimulatory effect could be blocked by PD123319 (Angiotensin II type 2 receptor (AT2R) inhibitor) and AG490 (Janus Kinase 2 (JAK2) inhibitor). As Pax2 is essential for the phenotypic conversion from mesenchymal stem cells to TECs during kidney development, we proposed that the re-expression of Pax2 in mature TECs may be an indicator of “atavistic” transition which mimics but reverses the processes of development of TECs. This could be proved by that a progenitor marker, CD24, was also found to be transiently expressed shortly after the expression of Pax2 in NRK-52E cells stimulated with Angiotensin II. The expression of CD24 was also suppressed by PD123319 and AG490. Moreover, knockdown of Pax2 by RNA interference could significantly reduce the expression of CD24 in NRK-52E cells stimulated with Angiotension II. Those findings suggest that mature TECs can trans-differentiate into progenitor-like cells by “atavistic transition”, which may participate in the recovery of tissue structure and Pax2 may play a pivotal role in this process. That might have important implications for further understanding of tubular regeneration after injury.

## Introduction

Acute kidney injury (AKI) is a common and severe clinical problem. The recovery of renal function after AKI depends on the recovery of renal tubular epithelium[1], but the mechanism of tubular epithelial reconstruction remains unclear. It has been proposed that surviving tubular epithelial cells (TECs) re-enter cell cycle and replace damaged TECs by proliferating, but the mechanism by which quiescent TECs regain the potential to regenerate is still unknown. Meanwhile, this model has been challenged by recent studies which suggest a role for stem/progenitor cells in kidney repair. Nevertheless, the origin of these stem/progenitor cells remains unclear [2], [3], [4], [5].

Our previous study demonstrated that TECs could be induced to temporarily re-express embryonic gene Paired box 2 (Pax2) during chronic kidney injury, which indicated that TECs could transform into an immature cell phenotype and participate in kidney repair during chronic kidney injury [6]. We then proposed that a similar "atavistic" phenotype transition might also occur during AKI [7]. This notion is supported by the finding that a mesenchymal cell marker, vimentin, could be expressed in tubular epithelium during the recovery stage of AKI [8].

The transition of TECs from one phenotype to another is not a new concept. During the embryonic stage of the kidney, the mesenchymal to epithelial transition (MET) which mesenchymal stem cells (MSCs) are converted into a polarized tubular epithelial phenotype is a pivotal event for the differentiation of TECs. Pax2 is expressed during MET and essential for this phenotype transition [9]. During chronic kidney injury, epithelial to mesenchymal transition (EMT) has been proven to occur in mature TECs. Pax2 is also expressed during EMT and essential for this phenotype transition [6]. Those findings suggest that the expression of Pax2 controls the phenotype transition between stem/progenitor cells and epithelial cells. Therefore, we believe that the re-expression of Pax2 in mature TECs may be a sign of cell atavistic transition, which mimics but reverses the genetic and cellular processes of tubular development. This atavistic transition allows mature TECs to gain a characteristic of stem/progenitor-like cells, which could re-differentiate into TECs that restore tissue structure.

In this study, we demonstrated that Pax2 was re-expressed in TECs during AKI in vivo, and we also found that Pax2 and a stem/progenitor cell marker, CD24, were temporarily re-expressed in NRK-52E cells stimulated with Angiotensin II (Ang II) in vitro. Both AT2R/JAK2 inhibitor and Pax2 iRNA could block the re-expression of Pax2 and CD24 in NRK-52E cells induced by Ang II. These findings led us to propose that mature TECs can undergo an atavistic transition to convert into stem/progenitor-like cells and participate in renal repair during AKI. Pax2 may play a central role in this process.

## Results

### The construction of AKI animal model

AKI was induced by ischemia-reperfusion injury (IRI) in Wistar rats as IRI is the most common cause of AKI [1]. The heart rate and glucose levels did not statistically differ before, during, or after operation both in experimental groups (IRI groups) and control groups (sham operation groups) (data not shown). The total elimination rate (due to death in all cases) was 5/96 (3/48 for IRI groups and 2/48 for sham operation groups).

**Figure 1:**
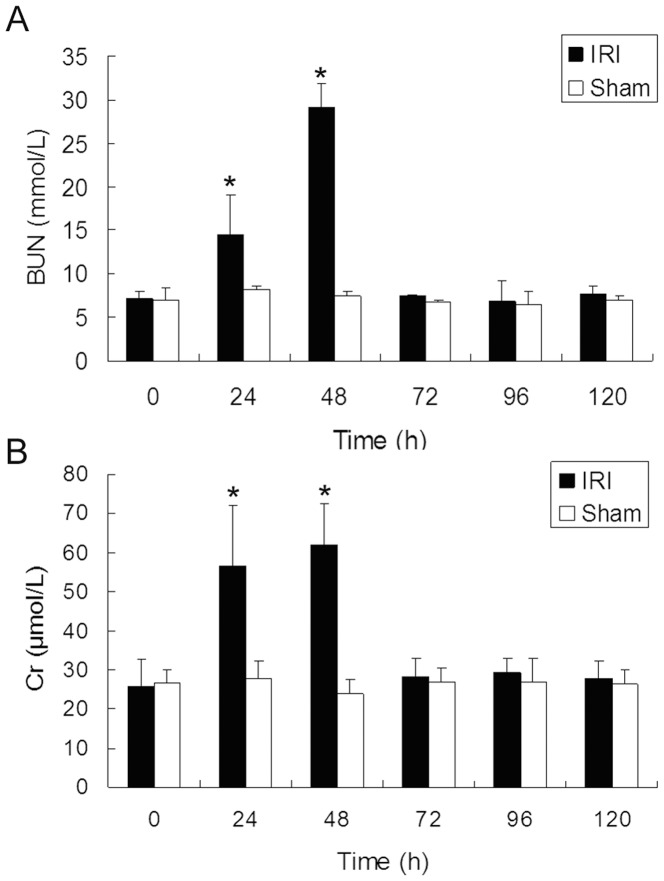
BUN (A) and Cr (B) results. In sham operation groups, there was no alteration of BUN and Cr levels after operation. In IRI groups, BUN and Cr levels increased significantly during the early stage of AKI (24 to 48 h after operation)(N = 6 for each time point, *P<0.05, IRI compared with sham), indicating impaired kidney function in IRI groups 24 to 48 h after operation. 72 h after operation, BUN and Cr levels in IRI groups decreased significantly and showed no difference with sham operation groups, indicating the recovery of renal function in IRI groups 72 h after operation.

The induction of renal damage by IRI was verified by biochemical and histological measurements. Both blood ureanitrogen (BUN) and creatinine (Cr) increased significantly during the early stage of AKI in IRI groups (24 to 48 hours (h) after operation). No alteration of BUN and Cr levels was observed in sham operation groups (figure 1).

**Figure 2:**
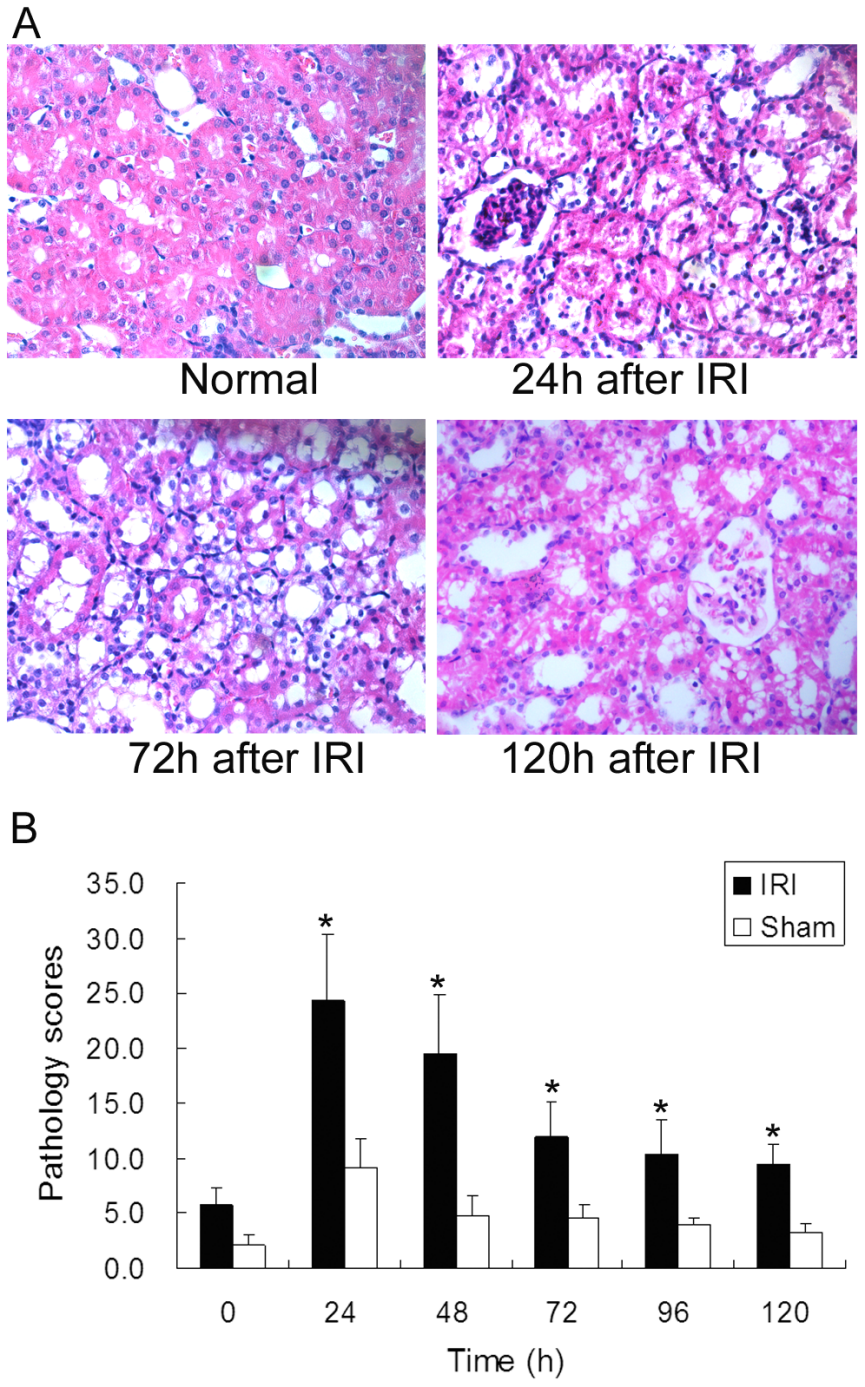
Pathology results of renal cortex sections. IRI groups showed remarkable changes on tubular structures detected by HE staining **(A)**. Disrupted brush borders and flattened epithelia were observed at 24 h after IRI, which indicated apparent tubular damage. Epithelial cell proliferation changes were observed at 72 h after IRI. Tissue structures recovery were observed at 120 h after IRI **(B)**. The pathology scores of the IRI groups significantly increased 24 h after IRI, and were sustained higher than sham operation groups 120 h after operation (*P<0.05, IRI compared with sham).

Kidney sections stained with hematoxylin-eosin (HE) showed remarkable changes consistent with AKI in IRI groups 24 h after IRI, such as disrupted brush borders and flattened epithelia. Epithelial cell proliferation changes were observed 72 h after IRI (figure 2A). Pathology scores of kidney showed significant increases after IRI in experimental groups, indicating remarkable renal tissue damages were induced by IRI (figure 2B). No significant changes were observed in animals of sham operation groups. Both the biochemical and histological results showed that the IRI-induced AKI rat model was successfully established.

### Re-expression of Pax2 in proximal tubular epithelium during recovery stage of AKI

**Figure 3:**
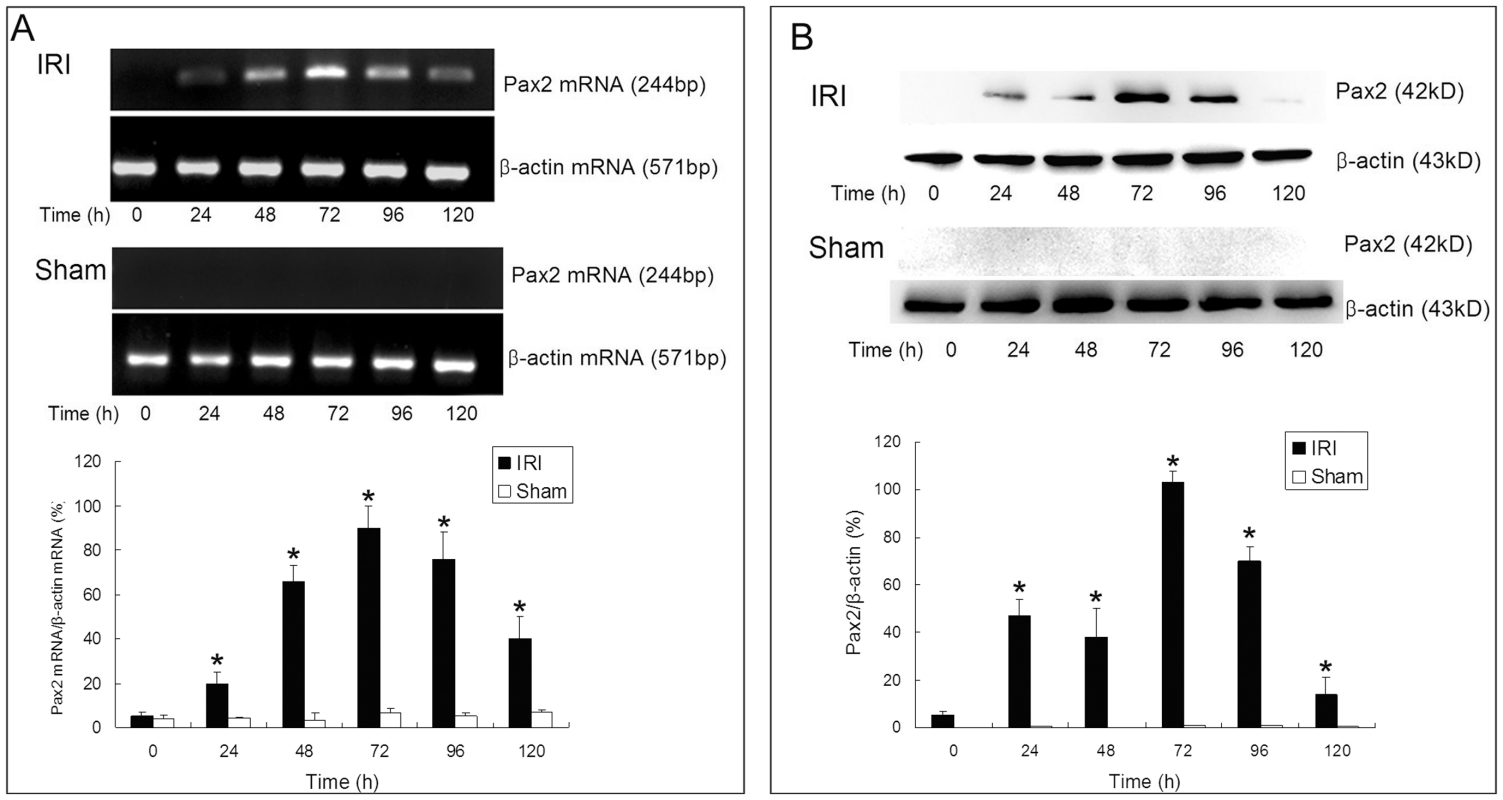
Pax2 expressions in renal cortex detected by RT-PCR and western blot. The mRNA **(A)** and protein **(B)** levels of Pax2 in renal cortex were detected via RT-PCR and western blot. Pax2 was expressed in the IRI groups 48–96 h after IRI operation (*P<0.05, IRI compared with sham), while Pax2 could not be detected in renal cortex of animals in sham-operation groups.

**Figure 4:**
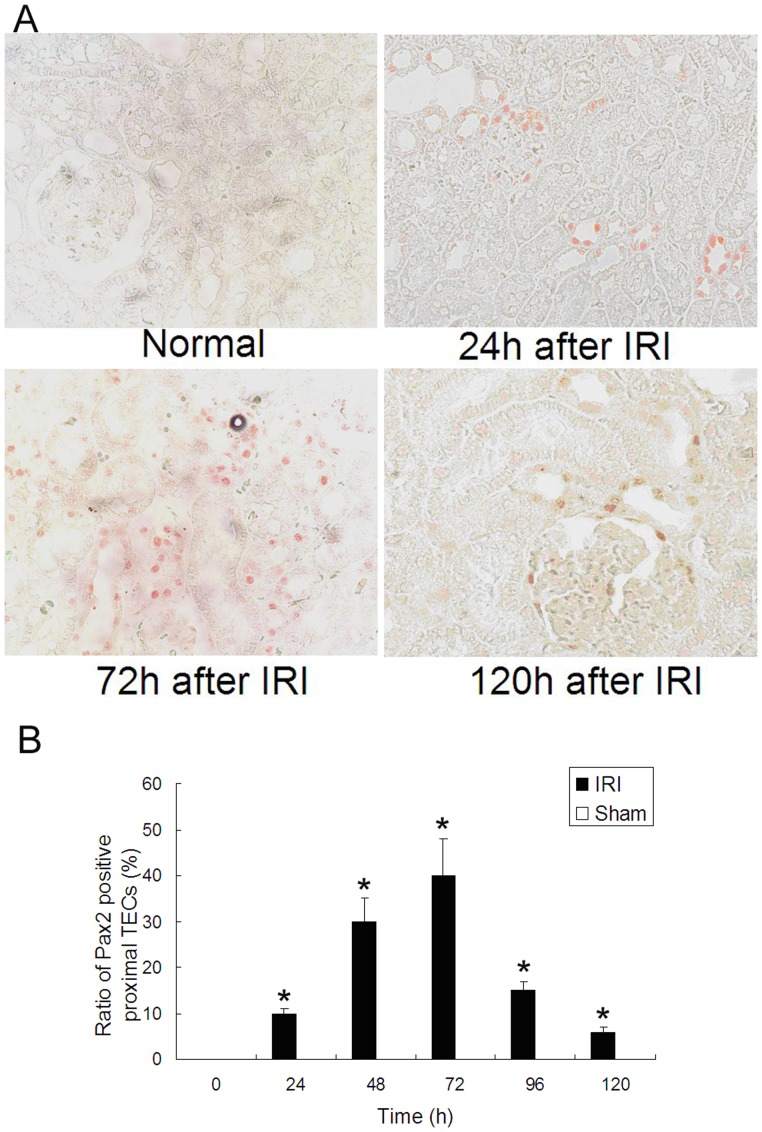
IHC detection of Pax2 in renal cortex. IHC detection showed that Pax2 was expressed in the nuclei of proximal TECs in the IRI groups 24–96 h after IRI **(A)**. The sham operation group did not show Pax2 expression in proximal tubules. The percentage of Pax2-positive proximal TECs increased 24 h after IRI and reduced to a low level 120 h after IRI, peaking at 72 h after IRI **(B)** (*P<0.05, IRI compared with sham).

The re-expression of Pax2 in the tubular epithelium during AKI was verified via reverse transcription polymerase chain reaction (RT-PCR), western blot and immunohistochemical (IHC) detections. A remarkable raise of Pax2 mRNA and protein levels was observed in the renal cortex of animals in IRI groups. The expression levels of Pax2 increased at 24 h, maximized at 72 h and decreased at 96 h after IRI. No apparent expression of Pax2 could be observed in sham operated animals (figure 3). The IHC detection showed that Pax2 could not be detected in proximal TECs in normal kidney, while 24-72 h after IRI, Pax2 was expressed in the nuclei of proximal TECs (figure 4). No changes of Pax2 expression pattern was observed in sham operation groups.

### Pax2 re-expression occurs primarily in injured epithelia cells

**Figure 5:**
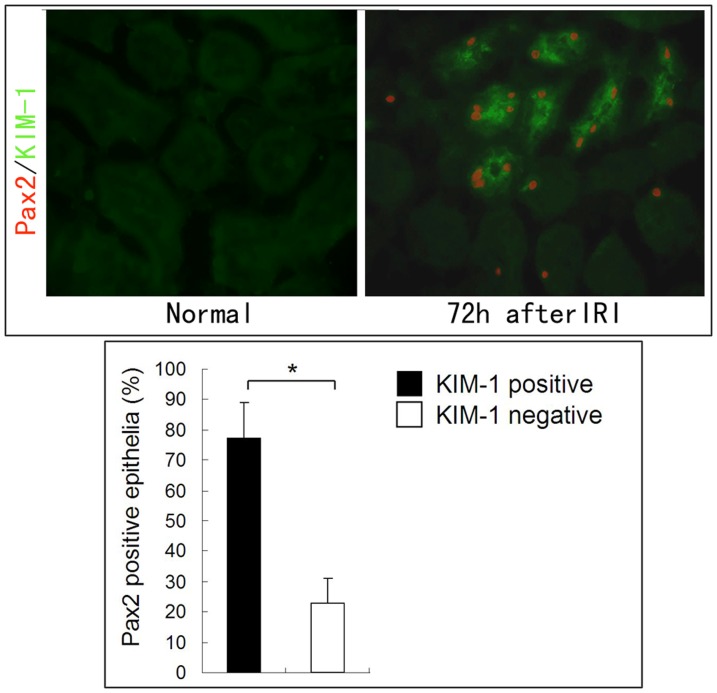
Pax2 is primarily expressed in injured proximal TECs. Uninjured kidneys showed no Pax2 and KIM-1 expression in proximal tubules. In IRI injured kidneys, there was 77.0±12.5% of Pax2-positive TECs co-expressed KIM-1(*P<0.05), indicating that injury induced proximal Pax2 was primarily expressed in injured proximal TECs.

To elucidate the relationship between Pax2 expression and tubular injury, we co-stained for Pax2 and kidney injury molecular-1 (KIM-1), a tubular injury marker, We found that 77.0±12.5% of Pax2-positive cells co-expressed KIM-1 (figure 5). This result indicates that most if not all Pax2 re-expression occur in injured TECs during AKI.

### Pax2 expression was accompanied by intrarenal Ang II increases in vivo

**Figure 6:**
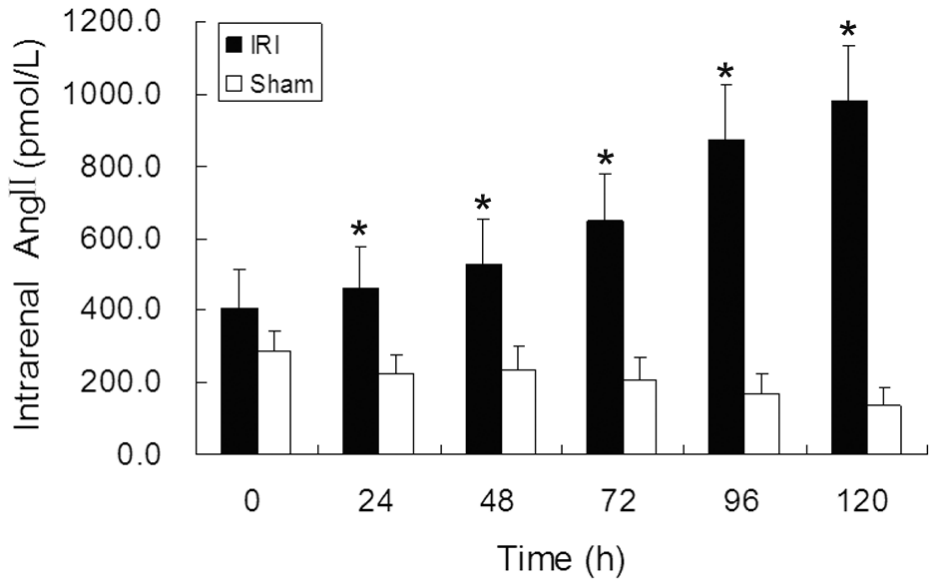
Intrarenal Ang II concentration in renal cortex. The levels of intrarenal Ang II in kidney cortex was detected by RIA. The IRI groups showed a significantly increase of Ang II after IRI operation while the sham groups showed no alteration. (*P<0.05, IRI compared with sham).

The intrarenal Ang II concentration significantly increased after operation and maintained a high level in IRI groups. The levels of intrarenal Ang II also slightly increased in sham operation groups and gradually decreased to a lower level thereafter (figure 6).

### Ang II stimulated Pax2 expression in NRK-52E cells via AT2R and JAK2

**Figure 7:**
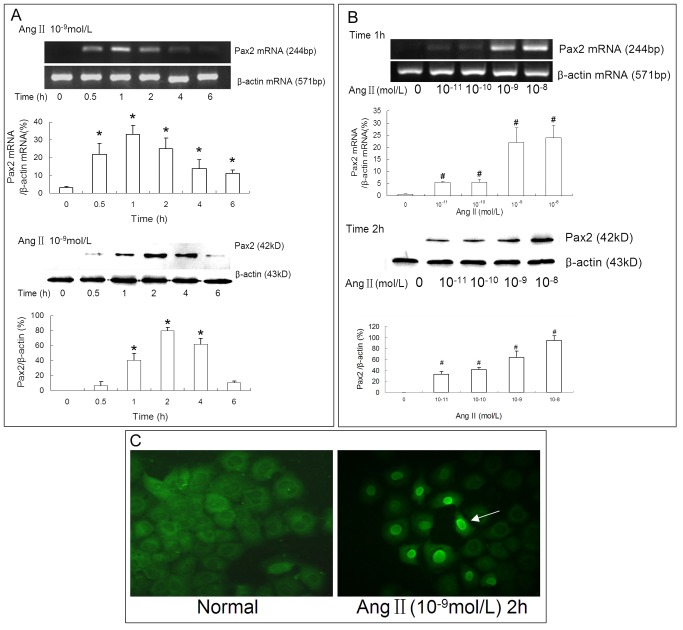
Ang II stimulates Pax2 expression in NRK-52E cells. RT-PCR and western blot data showed that Ang II could stimulate Pax2 expression in a time and dose dependent manner in NRK-52E cells; **(A)** Ang II at 10^−9^ mol/L stimulated Pax-2 expression in a time-dependent manner in NRK-52E cells, starting from 0.5 h post exposure and disappearing at 6 h. Pax2 mRNA level was maximized at 1 h and Pax2 protein level was maximized at 2 h. (*P<0.05, compared with Time = 0 h) **(B)** Ang II (10^−11^ mol/L to 10^−8^ mol/L) stimulated Pax-2 expression in a dose-dependent manner. The response to Ang II reaching a plateau at 10^−9^ mol/L. (#P<0.05 compared with Ang II  = 0 mol/L) **(C)** Immunofluorescence analyses showed that Pax2 was expressed in the nuclei of NRK-52E cells.

In vivo studies proved that the level of intrarenal Ang II increased during AKI and was accompanied by Pax2 re-expression. Therefore, we used Ang II to stimulate NRK-52E cells to verify that mature TECs have the ability of Pax2 re-expression. Pax2 was observed to be transiently expressed in NRK-52E cells in response to Ang II stimulation. Ang II stimulated Pax2 expression in a time- and dose-dependent manner (figure 7A, figure 7B). The immunofluorescence analyses showed that Pax2 was expressed in the nuclei of NRK-52E cells,which is consistent with the fact that Pax2 is a nuclear transcription factor (figure 7C).

**Figure 8:**
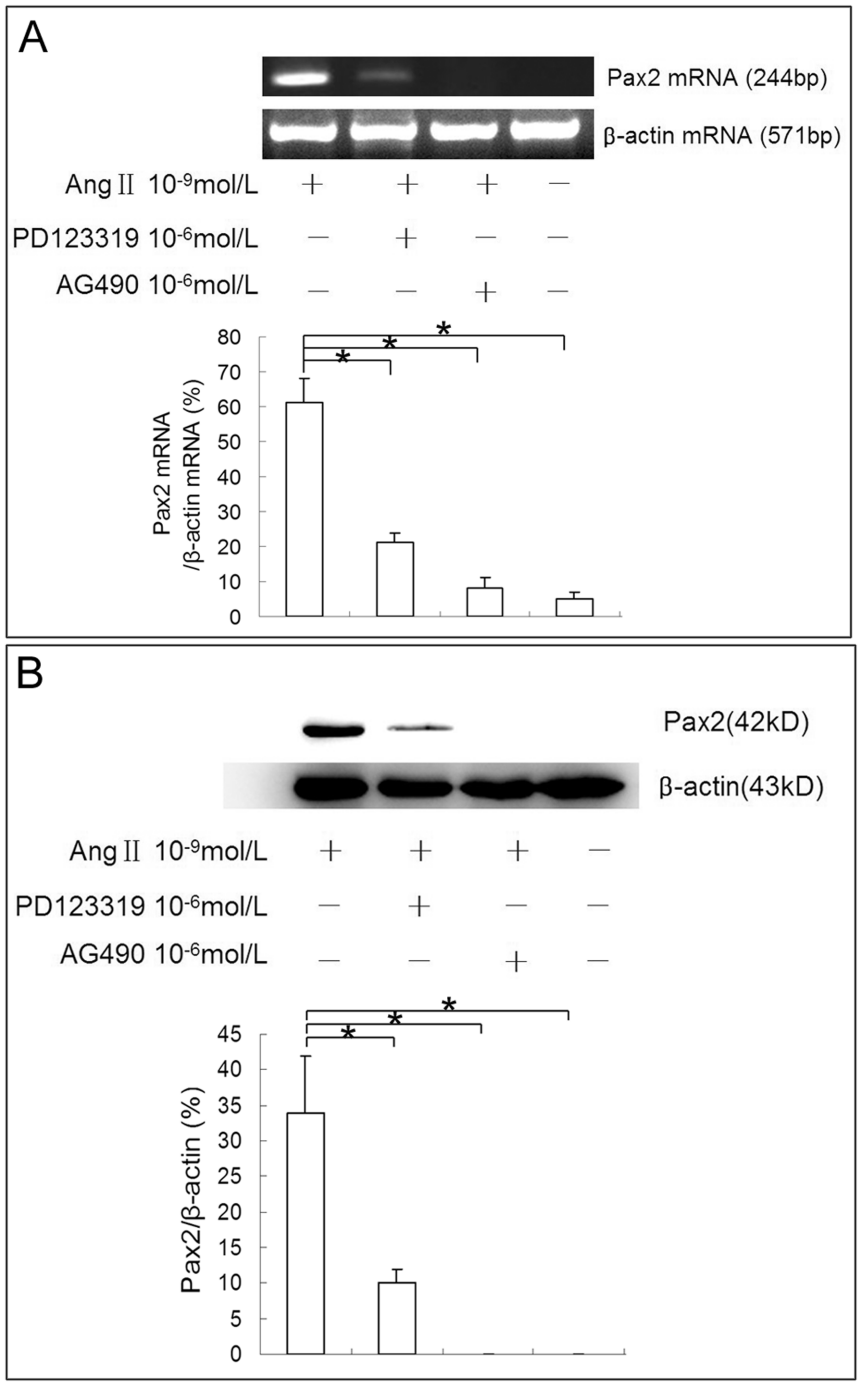
PD123319 and AG490 block the stimulatory effect of Ang II on Pax-2 expression in NRK-52E cells. NRK-52E cells were pretreated with PD123319 (10^−6^ mol/L) and AG490 (10^−6^ mol/L) in serum-free medium for 10 min before Ang II (10^−9^ mol/L) administration. Cells were harvest at 1 h (for RT-PCR analysis) and 2 h (for western blot analysis) post exposure. RT-PCR **(A)** and western blot **(B)** results showed that both AT2R blocker PD123319 and JAK2 blocker AG490 could block the stimulatory effect of Ang II on Pax2 expression in NRK-52E cells (*P<0.05).

To investigate signaling mechanisms that mediate Ang II induced Pax2 expression in NRK-52E cells, we pre-incubated NRK-52E cells before Ang II administration with PD123319 (AT2R inhibitor) and AG490 (JAK2 inhibitor). Results with western blot and RT-PCR showed that both PD123319 and AG490 could block the stimulatory effect of Ang II on Pax2 expression in NRK-52E cells (figure 8), suggesting that the stimulatory effect of Ang II on Pax2 expression in TECs is mediated by AT2R and JAK2 pathways.

These data indicate that mature TECs can re-express embryonic gene Pax2 in response to stimulation of injury factors like Ang II and the re-expression of Pax2 might be a sign of cell atavistic transition.

### Pax2 expression was accompanied by CD24 expression in NRK-52E cells induced by Ang II

**Figure 9:**
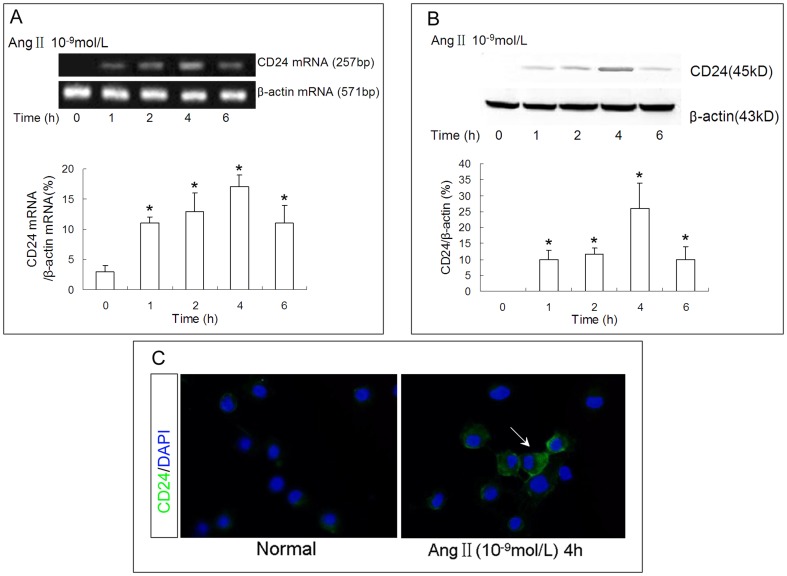
Ang II stimulates CD24 expression in NRK-52E cells. The RT-PCR **(A)** and western blot **(B)** data showed that 10^−9^ mol/L of Ang II could stimulate CD24 expression in NRK-52E cells shortly after the expression of Pax2; Ang II induced CD24 expression was started from 1 h post exposure, reached a peak at 4 h and began to disappear at 6 h (*P<0.05, compared with Time = 0 h). **(C)** Immunofluorescence analyses showed that CD24 was expressed at the surface of NRK-52E cells.

To further confirm that mature TECs have the ability of cell atavistic transition in response to stimulation of injury factors like Ang II, we measured the expression of a stem/progenitor cell marker, CD24[10], in NRK-52E cells stimulated by Ang II. We observed a transient expression of CD24 shortly after the expression of Pax2 in NRK-52E cells in response to Ang II stimulation. The immunofluorescence analyses showed that CD24 was expressed at the surface of NRK-52E cells, which consistent with the fact that CD24 is a cell membrane protein (figure 9).

**Figure 10:**
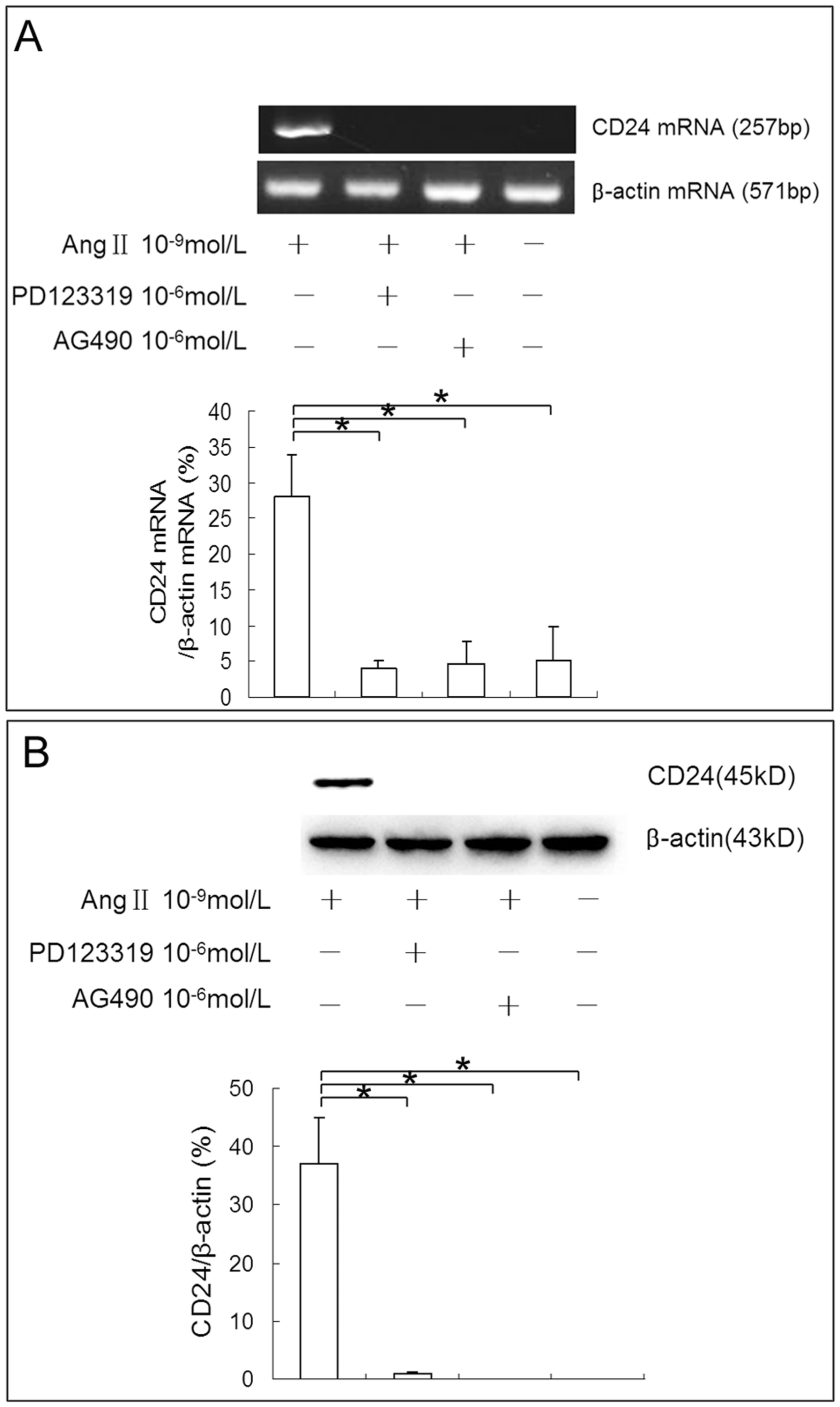
PD123319 and AG490 block Ang II induced CD24 expression in NRK-52E cells. NRK-52E cells were pretreated with PD123319 (10^−6^ mol/L) and the AG490 (10^−6^ mol/L) for 10 min before Ang II (10^−9^ mol/L) administration. Cells were harvest at 4 h post exposure for RT-PCR and western blot assays. Results of RT-PCR **(A)** and western blot **(B)** showed that both the AT2R blocker PD123319 and JAK2 blocker AG490 could block the stimulatory effect of Ang II on CD24 expression in NRK-52E cells (*P<0.05).

We also used PD123319 (AT2R inhibitor) and AG490 (JAK2 inhibitor) to examine whether the mechanisms that mediate Ang II induced CD24 expression are similar with that mediate Ang II induced Pax2 expression. Western blot and RT-PCR showed that both PD123319 and AG490 could inhibit Ang II induced CD24 expression (figure 10). These data suggest that Ang II stimulates the atavistic transition of TECs via the AT2R and JAK2 pathways.

### Knockdown of Pax2 suppressed Ang II induced CD24 expression in NRK-52E cells

The experiments above demonstrated that both Pax2 and CD24 could be stimulated in mature TECs by Ang II via the same signal pathways. Therefore, we used shRNA (pshRNA-Pax2-983)-mediated RNA interference of Pax2 to further determine the relationship between Pax2 and CD24 in NRK-52E cells induced by Ang II.

**Figure 11:**
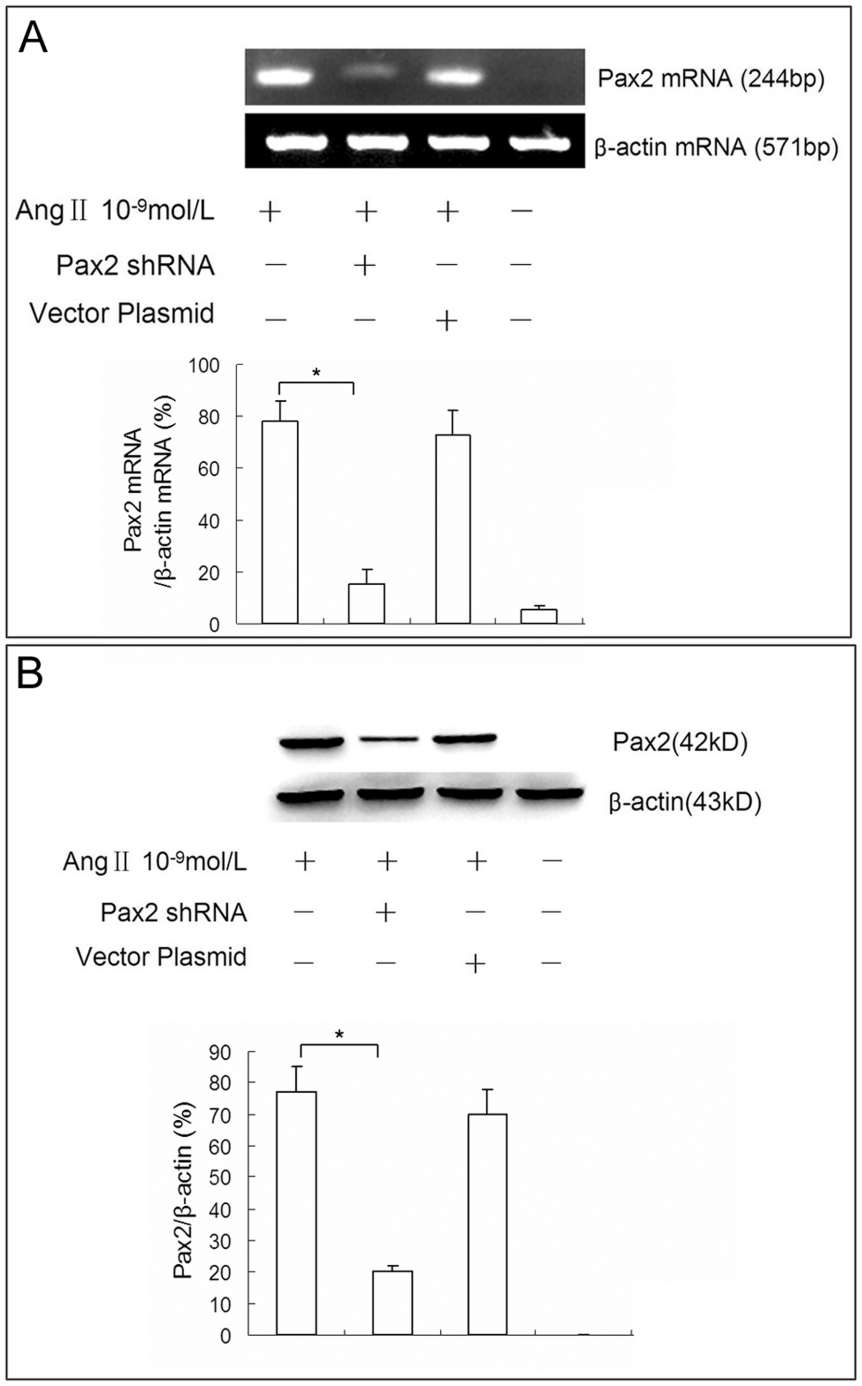
shRNA-mediated knockdown of Ang II induced Pax2 in NRK52E cells. NRK-52E cells were transfected with Pax2 shRNA or emptily plasmid by lipofection method before Ang II administration. RT-PCR **(A)** and western blot **(B)** data demonstrated that the transfection of NRK-52E cells with Pax2 shRNA resulted in a significant decrease in Ang II induced Pax2 expression. The repression rate was 74.3±8.2% as judged by western blot (*P<0.05). Transfection with emptily plasmid vector pGenesil-1 failed to reduce Pax2 expression. The effect of Pax2 shRNA was specific in that it failed to knock down the expression of unrelated protein β-actin.

Transfection of NRK-52E cells with Pax2 shRNA resulted in a significant decrease in Ang II induced Pax2 expression levels as shown by western blot and RT-PCR. The repression rate was 74.3±8.2% as judged by western blot. Transfection with emptily plasmid vector failed to reduce Pax2 expression. The effect of Pax2 shRNA was specific in that it failed to knock down the expression of unrelated protein β-actin (figure 11).

**Figure 12:**
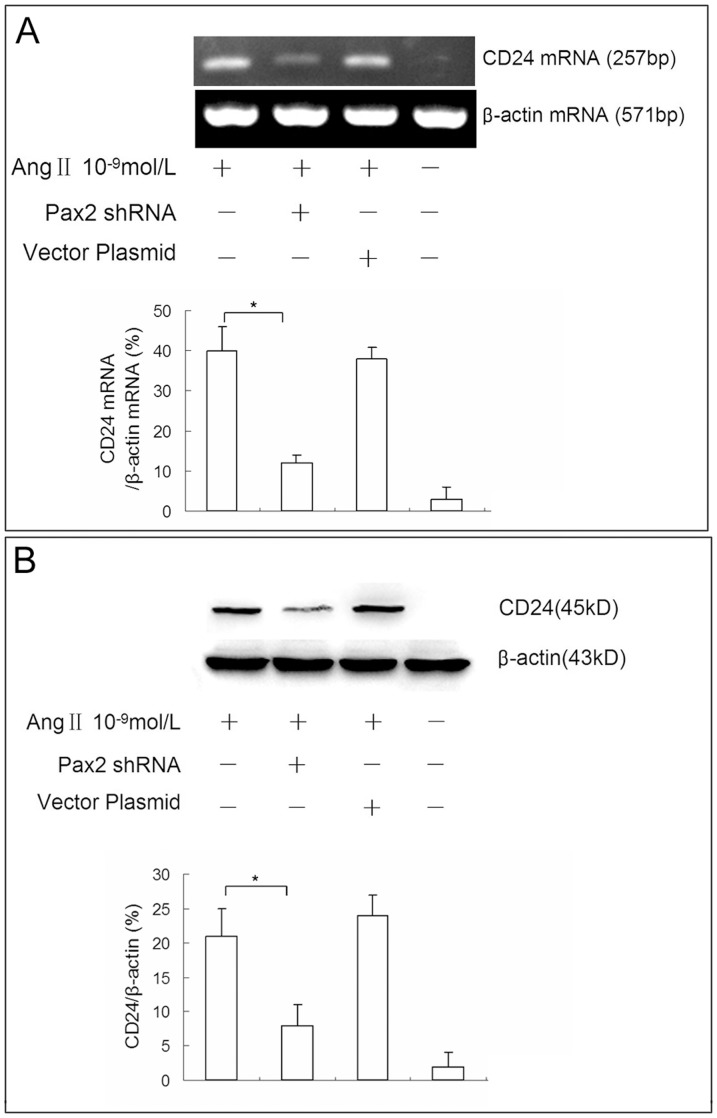
Knockdown of Pax2 suppressed Ang II induced CD24 expression in NRK52E cells. NRK-52E cells were transfected with Pax2 shRNA or emptily plasmid before Ang II administration. Cells were harvest at 4 h post exposure. RT-PCR **(A)** and western blot **(B)** results showed that knockdown of Pax2 significantly suppressed Ang II induced CD24 expression in NRK-52E cells, while transfection with emptily plasmid pGenesil-1 did not affect CD24 expression.

Western blot and RT-PCR showed that the knockdown of Pax2 significantly suppressed Ang II induced CD24 expression in NRK-52E cells (figure 12). These data suggest that Pax2 regulates the expression of CD24 in mature TECs induced by Ang II. That might indicate a central role of Pax2 in the cell atavistic transition process of TECs.

## Discussion

Pax2 is a transcription control factor which plays a crucial regulatory role during kidney development. During fetal life, Pax2 is essential for the mesenchymal to epithelial transition (MET), which mesenchymal stem cells (MSCs) are converted into tubular epithelial phenotype[9]. Pax2 is not originally expressed in MSCs, but is only induced when MSCs meet the ureteric bud. Pax-2 controls the process of MET by initiating a genetic cascade that includes WT1, Wnt4, Six2 and c-Ret to suppress the expression of mesenchymal markers, such as vimentin, and promote epithelial markers, such as E-cadherin and cytokeratin [11], [12], [13]. Pax2 is down-regulated once nephrogenesis is complete, showing a transient expression pattern [14].

Acute kidney injury (AKI) is a common and severe clinical problem. The recovery of renal tubular epithelium after AKI is critical for the recovery of renal structure and function. It is still not clear how quiescent TECs regain the potential to regenerate and whether a group of specialized stem/progenitor cells play a role during kidney repair [2], [3], [4]. Our previous study demonstrated that TECs could be induced to temporarily re-express Pax2 during chronic kidney injury [6], which suggested that mature TECs could transform into an immature cell phenotype during chronic kidney injury. We then proposed that a similar atavistic transition might also occur during AKI.

In this study, we observed a transient Pax2 re-expression in proximal tubular epithelium of IRI-induced AKI animals. Pax2 was expressed during the recovery stage of AKI. Most Pax2 positive epithelial cells co-expressed a tubular injury marker, KIM-1, indicating that Pax2 was primarily expressed in injured TECs. These data suggest that Pax2 re-expression might have a functional link with kidney epithelium injury and repair.

It remains to be shown which factors induce the transient re-expression of Pax2 in TECs after IRI. Intrarenal Angiotensin II (Ang II) is one of the most important injury factors in IRI-induced AKI [15], [16]. We verified a dramatic increase of intrarenal Ang II after IRI by RIA. Ang II plays an important role during tubular development and up-regulates Pax2 expression in fetal kidney epithelial cells [17], [18]. Thus, we hypothesized that Ang II might also stimulate the re-expression of Pax2 in mature TECs. We then used Ang II to simulate NRK-52E cells in vitro and verified that Ang II could stimulate Pax2 expression in mature TECs in a time- and dose-dependent manner. The expression of Pax2 in NRK-52Es also showed a transient pattern, which is consistent with the transient expression feature during tubular development.

During embryonic stage, the stimulatory effect of Ang II on Pax2 is mediated by AT2R. Pax2 and AT2R share a similar transient expression pattern during renal development, both of them are down-regulated in TECs when nephrogenesis is completed [17], [19], [20]. Interestingly, AT2R is also up-regulated in TECs after kidney injury [21], [22]. In fetal kidney epithelial cells, AT2R activates Pax2 via JAK2 pathway [18]. Therefore, we pre-incubated NRK-52E cells before Ang II administration with PD123319 (AT2R inhibitor) and AG490 (JAK2 inhibitor) to investigate signaling mechanisms that mediate Ang II induced Pax2 expression in NRK-52E cells. We found that both AT2R inhibitor and JAK2 inhibitor could block Ang II induced Pax2 expression in NRK-52E cells, indicating that the stimulatory effect of Ang II on Pax2 expression in mature TECs is mediated by AT2R-JAK2 pathway.

Renal Pax2 is down-regulated once nephrogenesis is complete and is not normally expressed in mature TECs. We believe that the re-expression of Pax2 in TECs is an initiating event of cell atavistic transition, which mimics but reverses the genetic and cellular processes of tubular development. This process could allow mature TECs to re-gain the characteristics of stem/progenitor cells. We provided evidence for this hypothesis by the observation that a progenitor marker, CD24 [10],was transiently expressed in Ang II induced NRK-52E cells shortly after the expression of Pax2. This model could also be supported by a previous study which showed that a mesenchymal cell marker, vimentin, was expressed in the tubular epithelium during the recovery stage of AKI[8].

Moreover, we found that both AT2R inhibitor (PD123319) and JAK2 inhibitor (AG490) which could not only block Ang II induced Pax2 expression, but CD24 expression as well in NRK-52E cells. This result indicaed a regulative role of Pax2 on CD24 expression. We then used shRNA-mediated RNA interference of Pax2 to further determine the relationship between Pax2 and CD24 in NRK-52E cells induced by Ang II. We found that knockdown of Pax2 expression resulted in an apparent suppress of Ang II induced CD24 expression in NRK-52E cells. These data demonstrate that CD24 is regulated by Pax2 in NRK-52E cells stimulated with Ang II.

In summary, our findings demonstrated that mature TECs can undergo an atavistic transition under the induction of acute injury factors such as Ang II. This atavistic transition allows mature TECs to re-gain the characteristics of stem/progenitor cells, which could re-differentiate into TECs to restore tissue structure. Pax2 might play a central part in this process. Currently, exogenous stem cells used for AKI treatment are difficult to obtain and extremely expensive. It is very likely that enhancing the atavistic transition of surviving TECs could be more effective than injecting exogenous stem cells. That might have important implications for further understanding of AKI treatment.

## Materials and Methods

### Animals and experimental model of AKI

The animal experiment was approved by the Medical Ethics Committee of the First Affiliated Hospital of Sun Yat-sen University. All animal experimental studies were conducted in accordance with the National Guidelines of Laboratory Animal use and Care in China. Male Wistar rats weighing 250 g to 300 g were obtained from the Experiment Animal Center of Sun Yat-sen University. Animals were maintained at a controlled temperature of 22±2°C with a 12 to 12 h light/dark cycle, with free access to food and water.

Animals were randomly divided into 12 groups of 6 animals per group. 6 groups received IRI operation and 6 groups received sham operation without IRI induction.

The rats were fasted overnight prior to the operation and anesthetized with an intraperitoneal injection of 10% freshly prepared chloral hydrate (350 mg/kg). Renal ischemia was induced by clamping both renal arteries with microserrefines. The clamps were removed after 45 min to re-establish blood flow in order to mimic the reperfusion injury. Sham operation groups received surgery without IRI induction. The body temperature was monitored with a rectal probe and maintained at 37°C during the entire procedure by surrounding the animals with 37°C water bags. The blood glucose levels and heart rate were measured before, during, and after the operation. After surgery, the incisions were sutured and rats were maintained in separated cages with free access to food and water.

Each group of rats was anesthetized with chloral hydrate at 0,24, 48, 72, 96, 120 h after operation. Blood samples were obtained by aspiration from abdominal vein. Blood ureanitrogen (BUN) and creatinine (Cr) levels were measured by a HITACHI 7170A Analyzer (HITACHI). The kidneys were removed and the renal cortex parts were separated for the subsequent experiments. The animals were then sacrificed by injecting overdose of chloral hydrate and potassium chloride.

Animals that did not show renal pathology changes or died after operation were excluded from the experimental groups.

### Histological studies

The cortex parts of kidneys were fixed in 10% neutral buffered formalin and embedded in paraffin; 2 μm thick sections were cut on a microtome (Rotationsmikrotom 3455 Leitz; Lecica), and hematoxylin-eosin (HE) staining was carried out to document the histological damage. Histological changes were examined via light microscopy in a blinded fashion according to the Pallers standard [23].

The immunohistochemical (IHC) studies were performed using an indirect immunoperoxidase technique: 4 μm thick sections were cut from the cortex sections of kidneys embedded in paraffin. The sections were de-paraffinized with xylene and rehydrated in graded ethanol. The endogenous peroxidase activity was quenched with 3% hydrogen peroxide for 10 min. The sections were heated in citrate buffer (pH 6.0) in a microwave oven at 1000 W for 15 min. After washing with 0.01 mol/L PBS, the sections were incubated with a 1%BSA blocking solution that contained 10% rabbit serum. After blocking, the sections were incubated with a 1:400 dilution of rabbit anti-Rat Pax2 polyclonal antibody (Santa Cruz) at 37°C for 1 h. After washing in PBS, the sections were covered with anti-rabbit secondary antibody (Cell Signaling Technology). The immunoreactivity was revealed using fast red (Biocare Medical) [24].

For double immuno-labeling, The cortex parts of kidneys were fixed in 4% paraformaldehyde and embedded in optimum cutting temperature compound. 10 μm thick frozen sections were cut on a Leica CM1850 UV frozen microtome (Leica). After washing in PBS, sections were permeabilized with 1% Triton ×100 for 5 min and antigens retrieved with heated citrate (pH 6.0) for 15 min. After blocking in blocking solution (5% goat serum and 0.3% Triton ×100 in PBS) at 37°C for 30 min, sections were incubated overnight at 4 °C with both rabbit anti-rat Pax2 polyclonal antibody (1∶200 dilution, Santa Cruz) and goat anti-rat KIM-1 polyclonal antibody (1∶400, B&D)(with 0.3% Triton ×100). Next, slides were washed in PBS and incubated for 2 h at 37°C in a mixture of FITC-conjugated anti-goat secondary antibody (1∶1000, Cell Signaling Technology) and APC-conjugated anti-rabbit secondary antibody (1∶1000, Cell Signaling Technology) (with 0.3% Triton ×100). The double labeling images were recorded with a fluorescence microscope (Nikon 80i Microscope) after washing with PBS.

### Radioimmunoassay for Angiotensin II

100 mg of fresh cortex sections of kidneys were frozen in liquid nitrogen and triturated using a mortar. 0.5 ml of Normal saline (NS) was added to each tissue sample. All samples were sonicated 4 to 5 times for 10 s each time and centrifuged at 15,000 rpm for 10 min at 4 °C.

The supernatants were reserved in order to measure the Ang II concentration using an Ang II 125 I radioimmunoassay kit (Buhlmann Laboratories). The assay was performed in the radioimmunoassay department of Sun Yat-sen University.

### Cell culture and treatment

The NRK-52E cell line was obtained from the American Type Culture Collection. Cells were grown in Dulbecco's modified Eagle's medium (DMEM)/F12 (Gibco) containing 10% FBS (Gibco) at 37°C in an atmosphere containing 5% CO_2_. Cells were synchronized overnight in serum-free medium before experiments. For Ang II stimulation experiment, cells were incubated in medium containing different concentrations of Ang II (10^−11^ mol/L to 10^−8^ mol/L). Cells were harvest at different time points post exposure (0.5–6 h for Pax2 and 1–6 h for CD24) for RT-PCR and western blot analysis. Cells incubated in serum free medium only were used as normal control. For immunofluorescence analysis, cells were treated similarly in 6 wells plates containing coverslips.

For signaling pathways study, cells were incubated with 10^−6^ mol/L AT2R blocker PD123319 or 10^−6^ mol/L JAK2 blocker AG490 for 10 min before Ang II (10^−9^ mol/L) administration. Cells incubated in serum free medium only were used as normal control. Cells incubated with Ang II (10^−9^ mol/L) without blockers were used as positive control. Cells were harvest at 1 h (for Pax2 RT-PCR), 2 h (for Pax2 western blot) and 4 h (for CD24 RT-PCR and western blot) after incubation.

### Immunofluorescence analysis

For the indirect immunofluorescence analysis, cells grown on coverslips were fixed with 4% para-formaldehyde for 20 min, followed by permeabilizing with 0.1% Triton X-100 for 30 min (For Pax2 detection). After blocking in 3% bovine serum albumin (BSA), cells were probed with each antibody as indicated. The following antibodies were used: rabbit anti-rat Pax2 polyclonal antibody (1∶400, Santa Cruz), rabbit anti-rat CD24 polyclonal antibody (1∶200, Santa Cruz) and FITC-labeled anti-rabbit secondary antibody (1∶1000, Cell Signaling Technology) [25]. The immunofluorescence images were recorded with a fluorescence microscope (Nikon 80i Microscope).

### Reverse transcription-PCR (RT–PCR)

Total RNA was extracted according to the manufacturer's protocol using Trizol Reagent (TaKaRa). The reverse transcription was performed using a ReverTra Ace-α cDNA Synthesis Kit (TOYOBO). PCR was performed in a 50 μl volume with TaKaRa PCR Kit (TaKaRa). The primers used for PCR are listed below: For Pax2, the forward primer was 5'-AACGGTGAGAAGAGGAAACG-3 and the reverse primer was 5'-GGAAGACATCGGGATAGGAA-3‘ (244 bp). For CD24, the forward primer was 5'-TCAAAAGTCACAACGGCAA-3' and the reverse primer was 5'- TCCAGTCCTCACATCCCAA-3' (257 bp). For β-actin, the forward primer was 5'-GCCCATCTATGAGGGTTAC-3' and the reverse primer was 5'-CTGGAAGGT GGACAGTGAG-3' (570 bp).

The reactions were initially heated to 95 °C for 3 min and then subjected to 30 cycles of 30 s at 94 °C, 30 s at 55°C, 30 s at 72 °C, and a final extension at 72°C for 10 min in a PCR Detection System (ABI 9700). The PCR products were separated by electrophoresis in 1.5% agarose gel.

### Western blot analysis

For western blot analysis, tissue samples were homogenized and sonicated prior to lysis. Cells and tissue samples were lysed for 1 h in a RIPA lysis buffer (50 mM Tris-HCl [pH 7.4], 150 mM NaCl, 1% NP-40,) in the presence of 1 μl/ml phenylmethylsulfonyl fluoride (PMSF). The lysed samples were centrifuged at 15,000 rpm for 20 min and boiled for 10 min. Protein concentrations were measured with a BCA Protein Assay Kit (Beyotime). Protein samples were separated via sodium dodecyl sulfate–polyacrylamide gel electrophoresis (SDS-PAGE) and electrotransferred onto a polyvinylidene difluoride (PVDF) membrane (Amersham Pharmacia Biotech). The PVDF membranes were blocked with TBST buffer (50 mM Tris-HCl [pH 7.6], 150 mM NaCl, 0.2% Tween 20) containing 5% non-fat dry milk for 0.5 h. After washing with TBST, the membranes were probed with each antibody as indicated. The following antibodies were used; rabbit anti-rat Pax2 polyclonal antibody (1∶400, Santa Cruz), rabbit anti-rat CD24 polyclonal antibody (1∶200, Santa Cruz), anti-mouse IgG and anti-rabbit IgG secondary antibody (1∶1000, Cell signaling technology). Immunoreactive proteins were visualized using an enhanced chemiluminescence (ECL) system (Forevergen). Triple replicates were performed for each experiment. The following antibodies were used: mouse anti-rat β-actin polyclonal antibody.

### RNA interference (RNAi) experiments

A previously designed short hairpin RNA (shRNA) pshRNA-Pax2-983 were used for the knockdown of Pax2 [6]. The sequence of pshRNA-Pax2-983 is 5'-GATCCGTGTGTCAGGCACACAGACGTTCAAGACG CGTCTGTGTGCCTGACACATTTTTTGTCGACA-3', which targeting at the 983-1002 bp of Pax2 coding region. The shRNA were sub-cloned into pGenesil-1 expression vector for transfection. The emptily vector pGenesil-1 was used as vector control. The plasmids were amplified and purified by Wuhan cell marker biotechnology Co., Ltd (Wuhan, China)

The RNAi experiment was conducted as previously described[6]. Briefly, cells were seeded 1 day before transfection at a density of 2×10^6^ cells/ml onto 35-mm plates in 2 ml of complete medium (DMEM/F-12 with 10% FBS). Cells were divided into 4 groups: Group A, serum-free medium alone; group B, Ang II (10^−9^ mol/L) stimulated; group C, shRNA and Ang II (transfected with pshRNA-Pax2-983 and stimulated with 10^−9^ mol/L of Ang II); group D, empty vector control (transfected with pGenesil-1 and stimulated with 10^−^ mol/L of Ang II).

Plasmid transfection was performed according to the protocol by Lipofectamine 2000 (Invitrogen). All cells were cultured in complete medium for 24 h after 4 h of transfection. Cells were then synchronized in serum-free medium fot 12 h. After that, cells in groups B, C and D were treated with 10^−9^ mol/L of Ang II and cells in group A were treated with serum-free medium alone. Cells were harvested at different time points for further experiments (1 h for RT-PCR of Pax2, 2 h for western blot of Pax2, 4 h for RT-PCR and western blot of CD24).

### Statistical Analyses

The data were expressed as Mean±SEM. The differences between groups were assessed by Student's t test. A p value < 0.05 was considered significant.
