# Correction: Cell Atavistic Transition: Paired Box 2 Re-Expression Occurs in Mature Tubular Epithelial Cells during Acute Kidney Injury and Is Regulated by Angiotensin II

**DOI:** 10.1371/journal.pone.0127718

**Published:** 2015-05-06

**Authors:** Yushen Jiang, Tang Jiang, Juan Ouyang, Qingsong Zhou, Yanlan Liang, Yingpeng Cui, Peisong Chen, Bin Huang

In Fig 3A of the original published article the panels for beta-actin mRNA were duplicated for the Sham and IRI treatment. The authors apologize for this error and have provided the correct figure here.

**Fig 3:**
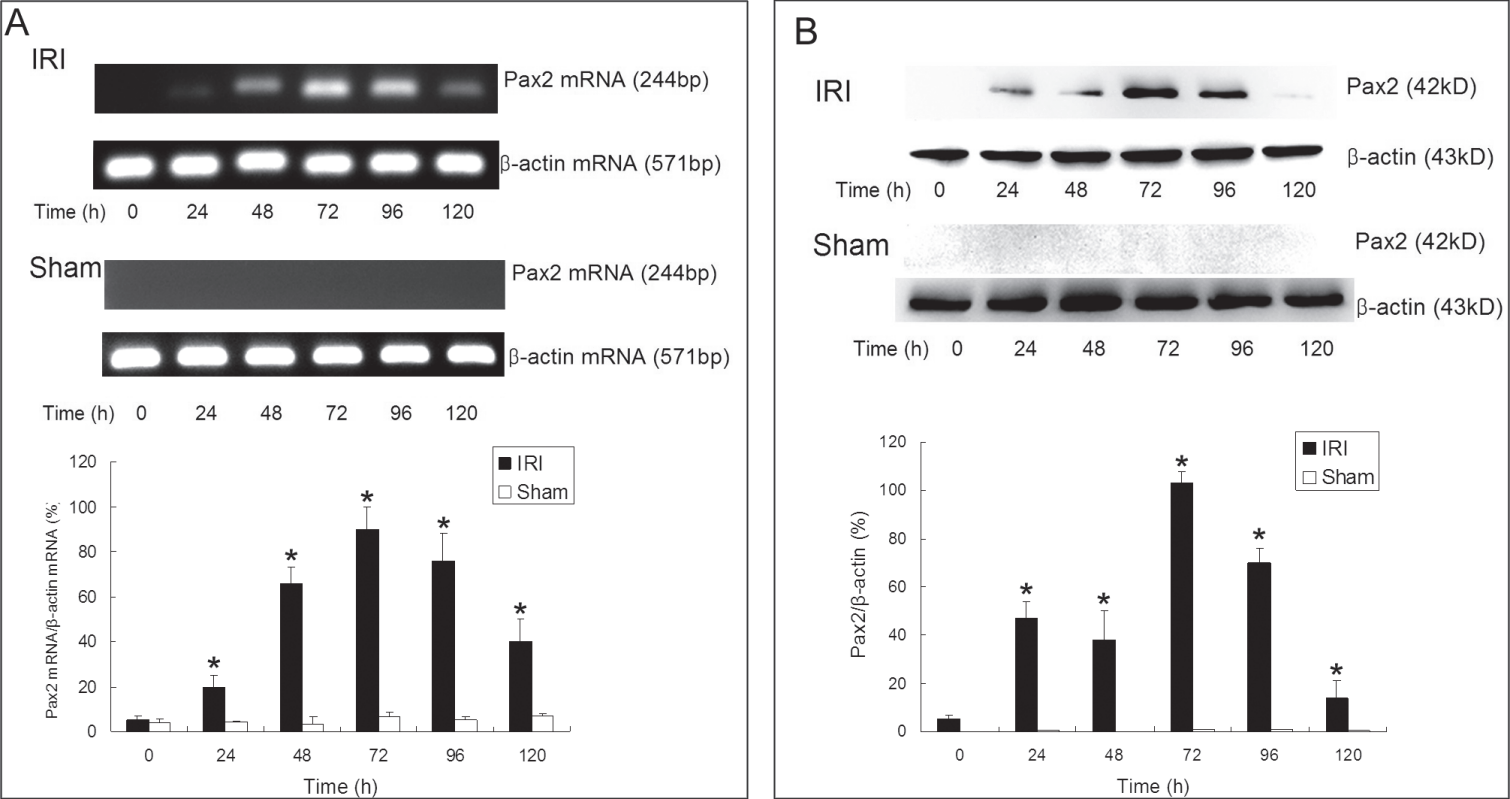
Pax2 expressions in renal cortex detected by RT-PCR and western blot. The mRNA **(A)** and protein **(B)** levels of Pax2 in renal cortex were detected via RT-PCR and western blot. Pax2 was expressed in the IRI groups 48–96 h after IRI operation (*P<0.05, IRI compared with sham), while Pax2 could not be detected in renal cortex of animals in sham-operation groups.
